# Right hemihepatectomy combined with ligation of the common hepatic artery and gastroduodenal artery for the treatment of intrahepatic HHT: A case report

**DOI:** 10.3389/fsurg.2022.900297

**Published:** 2022-08-09

**Authors:** Jifeng Xiang, Wei Xie, Cuncheng Zhang, Huaizhi Wang

**Affiliations:** ^1^ Institute of Hepatopancreatobiliary Surgery, Chongqing General Hospital, Chongqing, China; ^2^ Department of Ultrasonography, Chongqing General Hospital, Chongqing, China

**Keywords:** hereditary haemorrhagic telangiectasia, right hemihepatectomy, common hepatic artery, gastroduodenal artery, ligation

## Abstract

Hereditary haemorrhagic telangiectasia (HHT) is a rare disease that lacks effective treatment. Here, the authors report the case of a 30-year-old woman presenting with abdominal pain accompanied by severe malnutrition. After a definite diagnosis of HHT involvement in the liver, liver transplantation was the first-choice treatment according to the guidelines of HHT. However, the patient firmly refused liver transplantation. Finally, a new type of surgery, right hemihepatectomy combined with ligation of the common hepatic artery and gastroduodenal artery, was performed based on careful study of the case and with the maximum benefit of the patient in mind. Although the patient developed transient liver dysfunction after surgery, she eventually recovered well and continued to be followed up. As far as we know, this is the first report of this kind of surgery for the treatment of intrahepatic HHT.

## Introduction

Hereditary haemorrhagic telangiectasia (HHT) is a rare autosomal dominant disorder characterized by arteriovenous malformations (AVMs) of the internal organs and mucocutaneous telangiectasias (1). The estimated prevalence is 1/5,000–1/8,000 people (2). The liver is the most common visceral organ susceptible to HHT, but such cases are usually asymptomatic. More than half of patients with HHT have hepatic vascular malformations (VMs), but only 8% show symptoms (3). Although asymptomatic liver VMs do not require treatment, symptomatic liver VMs must be treated aggressively since they increase mortality. However, the treatment for liver VMs is limited to anti-angiogenesis, arterial embolization, and ligation and banding of the hepatic artery. Liver transplantation is an effective treatment option, whereas long-term immunosuppressive therapy remains a trade-off of this choice, especially for younger patients. Therefore, more effective treatments for liver VMs should be explored. Here, we report a 30-year-old woman who had HHT with hepatic AVMs and underwent right hemihepatectomy combined with ligation of the common hepatic artery and gastroduodenal artery because of her disease characteristics. Although long-term outcomes require further follow-up, the successful recovery and discharge of the patient demonstrate the feasibility of this procedure for the treatment of partial HHT with hepatic AVMs.

## Case description

**Figure 1:**
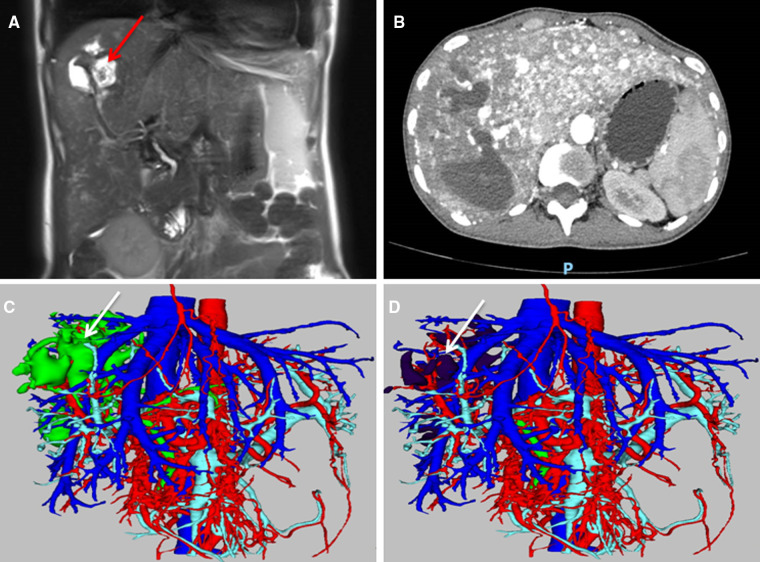
Imaging of HHT involvement in the liver. (**A**) The MRI results suggested right hepatobiliary dilatation with stone formation (arrow); (**B**) The arterial phase of CT indicated diffuse telangiectasia and shunting in the liver; (**C**) Three-dimensional reconstruction showed hepatic telangiectasia and bile duct dilatation (arrow); (**D**) Three-dimensional reconstruction showed telangiectasia and intrahepatic bile duct stones (arrow).

A 30-year-old woman was admitted to our hospital with abdominal pain and weight loss. The patient had a history of recurrent epistaxis during pregnancy. The patient had no family history of HHT. Physical examination showed tenderness in the upper abdomen. Laboratory results on admission showed moderate anaemia, abnormal liver function and severe malnutrition. Abdominal ultrasonography showed disorder of the intrahepatic duct structure. Magnetic resonance imaging (MRI) showed that intrahepatic bile duct stones were concentrated in the right hemi-liver (Figure 1A). Moreover, hepatic arteriovenous malformations (AVMs) accompanied by bile duct necrosis were identified by computed tomography (CT) (Figure 1B). Finally, three-dimensional reconstruction vividly showed the dilatation of the intrahepatic bile ducts, bile duct calculi, and extensive telangiectasia (Figures 1C, D). Fortunately, cerebral angiography, thoracic and abdominal angiography, gastroscopy, colonoscopy, echocardiography and cardiac biomarker examination showed no obvious abnormalities. According to the Curaçao diagnostic criteria for HHT (4) (**Supplementary Table S1**), she was diagnosed with HHT with hepatic AVM.

After diagnosis, the treatment was discussed by a multidisciplinary team, and liver transplantation was deemed one of the best treatment options for this patient according to international guidelines (5). The patient refused liver transplantation, so alternative treatments had to be considered to relieve her abdominal pain symptoms and control her biliary tract infection. Based on the characteristics of the patient, although hepatic AVMs were widespread throughout the liver, the major lesions (bile duct stones and biliary tract infection) were localized to the right hemi-liver. Finally, right hemihepatectomy combined with ligation of the common hepatic artery and gastroduodenal artery was planned. Since ligation of the common hepatic artery and gastroduodenal artery may effectively alleviate the hepatic arteriovenous shunt (6, 7), a right hemihepatectomy was expected to eradicate the bile duct stones and the infected lesion. To achieve optimal treatment outcomes, the patient spent two months on preoperative preparation, which included improving her nutritional status, gaining weight, getting cardiorespiratory exercise, etc.

**Figure 2:**
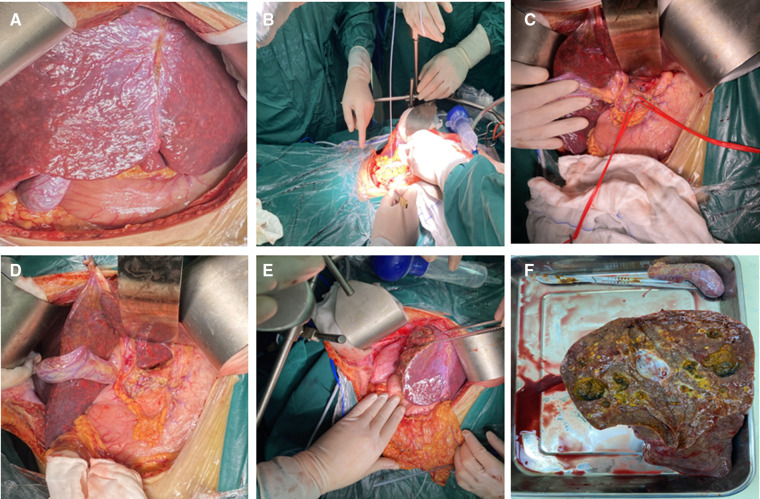
The operative procedure and key intraoperative pictures. (**A**) Surgical exploration revealed extensive telangiectasia in the liver; (**B**) Portal venous pressure measurement prior to the surgery; (**C**) The common hepatic artery and gastroduodenal artery were suspended and pre-occluded; (**D**), The common hepatic artery and gastroduodenal artery were ligated; (**E**) Right hemihepatectomy was performed successfully; (**F**) Right liver specimen and intrahepatic bile duct dilatation with stone formation.

**Figure 3:**
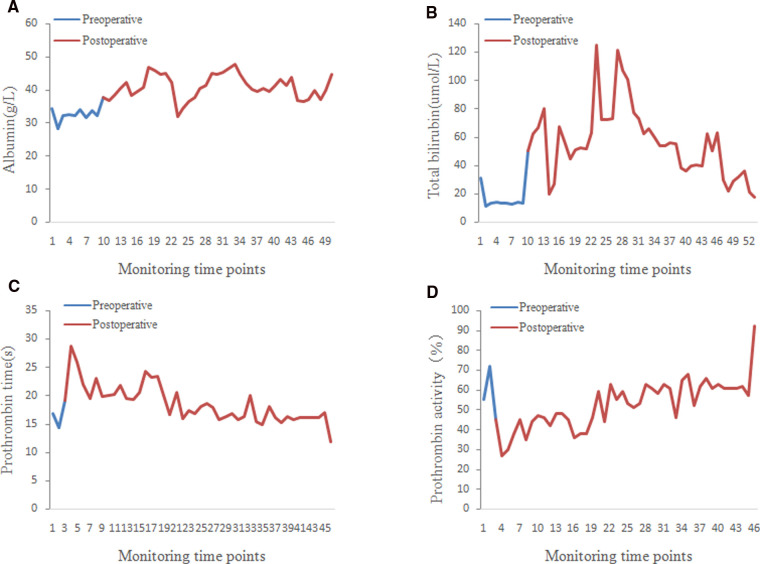
Curve of postoperative liver function-related indexes. Postoperative changes in albumin (**A**) total bilirubin (**B**) prothrombin time (**C**) and prothrombin activity (**D**).

**Figure 4:**
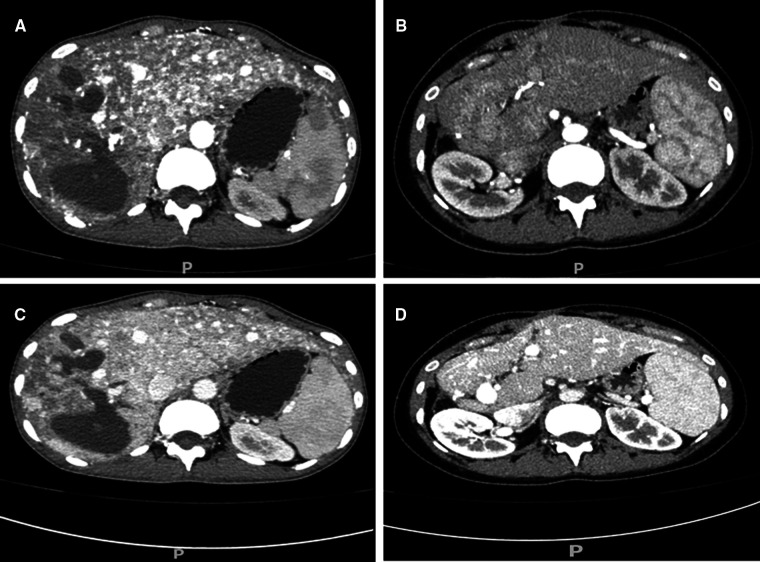
CT image comparison of the hepatic arteriovenous shunt before and after operation. CT arterial phase showed a significant decrease in telangiectasia and the shunt (**A**, preoperative; **B**, postoperative). Enhanced CT in the portal phase also showed a significant decrease in the shunt (**C**, preoperative; **D**, postoperative).

On intraoperative exploration, the erythema caused by telangiectasia was diffuse throughout the liver, which was consistent with the imaging findings (Figure 2A). Despite careful preoperative preparation, the operation was still very risky. Therefore, several measures were taken to reduce surgical complications:1, portal pressure was monitored to ensure that the portal pressure was reduced while normal perfusion was maintained (Figure 2B); 2, the blood flow velocities of the hepatic artery, portal vein and hepatic vein were monitored by ultrasound to ensure nearly normal blood flow signal after ligation (Figures 2C,D; **Supplementary Table S2**); and 3, since all indicators met our expectations, right hemihepatectomy was performed as the last step (Figures 2E,F). In the end, the surgery was successful. As expected, even with her extensive preoperative preparation, there was a period of liver insufficiency after surgery (Figure 3). Importantly, the patient recovered thanks to aggressive supportive treatment. The patient has been followed up regularly (every 6 months, including enhanced upper abdominal CT and liver function), and the treatment effect was satisfactory (pain disappeared, liver function returned, weight increased, liver volume was partially compensated for, and hepatic AVMs were relieved) (Figure 4).

## Discussion

HHT is a rare autosomal dominant disease characterized by organ telangiectasia. The liver is the most commonly affected organ (hepatic AVMs) (8); however, the treatment options are limited and unsatisfactory. Although the vast majority of liver VMs are asymptomatic and do not require treatment, symptomatic liver VMs must be treated aggressively since they increase mortality (9). Intravenous bevacizumab helps relieve symptoms and has been used as a bridge to orthotopic liver transplant (OLT) (10). Given that hepatic artery embolization is associated with mortality, it is only recommended in late-stage high-output cardiac failure (HOCF), only when medical therapies fail, and only when OLT is not an option (5). Moreover, embolization is contraindicated in patients with cholangitis or bile duct necrosis (11). Hepatic artery ligation or banding is considered to be effective in the treatment of HOCF and hepatic arteriovenous shunt in certain patients (6, 7, 12). However, it would not have been enough to cure the disease of this patient, who had bile duct stones and cholangitis in the right hemi-liver. Liver transplantation is considered to be the most effective treatment for hepatic AVMs, but this comes along with life-long immunosuppressive treatment to reduce the risk of organ rejection (13).

This young patient had HHT with liver involvement accompanied by bile duct stones and cholangitis. She refused liver transplantation. Fortunately, in addition to the diffuseness of her hepatic AVMs, her biliary tract lesions were localized to the right hemi-liver. Therefore, we designed right hemihepatectomy combined with ligation of the common hepatic artery and gastroduodenal artery based on the individual characteristics of this patient. Lesion resection for arteriovenous malformations has been previously reported, but right hemihepatectomy combined with artery ligation for the treatment of HHT has not. Georghiou et al. successfully cured pulmonary arteriovenous malformation by lobectomy (14). A recent report suggested that partial liver resection is also an option for large intrahepatic shunts that do not close spontaneously (15). Petrovic et al. reported a successful left lateral bisegmentectomy in a HHT patient with liver abscess (16). These reports provided a rationale for hepatectomy, as long as the patient's residual liver is sufficient. We also performed ligation of the common hepatic artery to alleviate the AVMs, while ligation of the gastroduodenal artery helped to avoid collateral circulation. It is important to emphasize that we deliberately kept the hepatic artery and portal vein pressure slightly higher than normal during the operation to maintain hepatic blood perfusion. As expected, although the patient developed transient liver dysfunction after surgery, she was discharged from the hospital in good health.

In the face of such a rare disease as HHT, available treatment options are limited, and their effect is unsatisfactory. However, for some special cases, we may still be able to design a relatively good individualized treatment other than the traditional treatment and protect the best interests of the patient. The successful treatment of this patient indicates that ligation of the common hepatic artery and gastroduodenal artery combined with hepatectomy is suitable for some HHT patients with localized intrahepatic lesions. This patient's success further broadens the treatment options for HHT.

## Data Availability

The original contributions presented in the study are included in the article/**Supplementary Material**, further inquiries can be directed to the corresponding author/s.
