# Supplementary material for: Right hemihepatectomy combined with ligation of the common hepatic artery and gastroduodenal artery for the treatment of intrahepatic HHT: A case report
Source: Front Surg. 2022 Aug 9;9:900297. doi: 10.3389/fsurg.2022.900297 (PMC9395736; doi:10.3389/fsurg.2022.900297)
Supplement: Supplementary file 1 [file Table_1_v1.docx]

Suppl. Table 1 The Curaçao criteria

| Criteria | Description |
| --- | --- |
| 1.Epistaxis | spontaneous, recurrent nose bleeds |
| 2.Telangiectases | multiple, at sites: lips, oral cavity, fingers and nose |
| 3.Visceral lesions | Gastrointestinal telangiectasia (with or without bleeding), Pulmonary AVM, Hepatic AVM, Cerebral AVMs, Spinal AVM |
| 4.Family history | a first degree relative with HHT according to these criteria |

A diagnosis of HHT is considered ‘definite’ if three or more criteria are present, ‘possible or suspected’ if two criteria are present, and ‘unlikely’ if 0 or 1 criterion is present
