# Supplementary material for: Right hemihepatectomy combined with ligation of the common hepatic artery and gastroduodenal artery for the treatment of intrahepatic HHT: A case report
Source: Front Surg. 2022 Aug 9;9:900297. doi: 10.3389/fsurg.2022.900297 (PMC9395736; doi:10.3389/fsurg.2022.900297)
Supplement: Supplementary file 2 [file Table_2_v1.docx]

Suppl. Table 2 Changes in hepatic haemodynamics before and after arterial ligation

|  | Before ligation of the artery | After ligation of the artery |
| --- | --- | --- |
| Portal pressure (cmH2O) | 19 | 10 |
| Hepatic artery velocity (cm/s) | 300 | 140 |
| Portal velocity (cm/s) | 70 | 45 |
| Hepatic venous velocity (cm/s) | 70 | 50 |
| Vena cava velocity (cm/s) | 75 | 70 |
